# Factors of Force Potentiation Induced by Stretch-Shortening Cycle in Plantarflexors

**DOI:** 10.1371/journal.pone.0120579

**Published:** 2015-06-01

**Authors:** Atsuki Fukutani, Toshiyuki Kurihara, Tadao Isaka

**Affiliations:** 1 Research Organization of Science and Technology, Ritsumeikan University, Kusatsu, Shiga, Japan; 2 Japan Society for the Promotion of Science, Research Fellowship for Young Scientists, Chiyoda-ku, Tokyo, Japan; 3 Faculty of Sport and Health Science, Ritsumeikan University, Kusatsu, Shiga, Japan; Duke University, UNITED STATES

## Abstract

Muscle force is potentiated by countermovement; this phenomenon is called stretch-shortening cycle (SSC) effect. In this study, we examined the factors strongly related to SSC effect in vivo, focusing on tendon elongation, preactivation, and residual force enhancement. Twelve healthy men participated in this study. Ankle joint angle was passively moved by a dynamometer, with a range of motion from 15° dorsiflexion (DF) to 15° plantarflexion (PF). Muscle contraction was evoked by electrical stimulation, with stimulation timing adjusted to elicit three types of contraction: (1) concentric contraction without preliminary contraction (CON), (2) concentric contraction after preliminary eccentric contraction (ECC), and (3) concentric contraction after preliminary isometric contraction (ISO). Joint torque was recorded at DF5°, PF0°, and PF5°, respectively. SSC effect was calculated as the ratio of joint torque obtained in ECC or ISO with respect to that obtained in CON at the aforementioned three joint angles. SSC effect was prominent in the first half of movement in both ECC (DF5°, 329.3 ± 101.2%; PF0°, 159.2 ± 29.4%; PF5°, 125.5 ± 20.8%) and ISO (DF5°, 276.4 ± 87.0%; PF0°, 134.5 ± 24.5%; PF5°, 106.8 ± 18.0%) conditions. SSC effect was significantly larger in ECC than in ISO at all joint angles (*P* < 0.001). Even without preliminary eccentric contraction (i.e., ISO condition), SSC effect was clearly large, indicating that a significant part of SSC effect is derived from preactivation. However, the active lengthening-induced force potentiation mechanism (residual force enhancement) also contributes to SSC effect.

## Introduction

Muscle force is potentiated by preliminary eccentric contraction; this phenomenon is widely known as stretch-shortening cycle (SSC) [1, 2]. Since SSC is a very well-known phenomenon, numerous studies have been conducted on this topic [3, 4]. Based on the results of these previous studies, possible mechanisms of force enhancement by SSC (SSC effect) have been proposed, including stretch reflex [5, 6], tendon elongation [7, 8, 9], preactivation [10, 11, 12], and residual force enhancement [13, 14, 15]. Residual force enhancement is also referred to as “potentiation” or “potentiation of the contractile machinery” [14, 16].

Recently, several studies have examined the factors strongly related to SSC effect. For example, Walshe et al. [17] reported that preactivation and potentiation (i.e., residual force enhancement) were related to SSC effect. In addition, Hirayama et al. [18] reported that tendon elongation was strongly related to SSC effect, whereas Zajac [19] and Bobbert et al. [11] suggested that the contribution of tendon elongation was not large. These relatively ambiguous results among studies may be caused by the difficulty in extracting each possible factor separately. For example, when we examine SSC effect by comparing jump height of a rebound jump (SSC) with that of a squat jump (no SSC), it is easy to confirm whether or not jump height increases by SSC. On the other hand, it is difficult to determine which factors contribute to SSC effect and/or the extent of contribution of each factor, because some factors change simultaneously. Therefore, to clarify the mechanism of SSC effect, each factor should be extracted separately by controlling the experimental conditions. However, especially in human experiments adopting rebound and squat jumps, controlling activation level of muscle, range of motion, and/or angular velocity among trials is difficult, which can affect interpretation of the results. To overcome these difficulties, an experimental design with single-joint motion regulated by a dynamometer, such as the experiment conducted by Finni et al. [7], would be useful because range of motion and angular velocity can be well controlled by the dynamometer. In addition, adopting electrically-evoked contraction rather than voluntary contraction would be beneficial because activation level of muscle can be set identical among trials by controlling electrical stimulation parameters.

Therefore, the purpose of this study was to examine in detail the factors that contribute to SSC effect by using the aforementioned experimental setup. To accomplish this purpose, the influence of preactivation was isolated by adopting isometric contraction as a preliminary contraction conducted before concentric contraction. Because there was no active lengthening phase in this trial, the contribution of tendon elongation and residual force enhancement can be discarded, whereas the contribution of preactivation was included. Thus, if SSC effect was similar between concentric contraction following isometric contraction condition and concentric contraction following eccentric contraction condition, the influence of tendon elongation and/or residual force enhancement would be small or negligible. On the other hand, if SSC effect was different between the above two conditions, tendon elongation and/or residual force enhancement should have a substantial influence on SSC effect. We hypothesized that SSC effect is larger in concentric contraction following eccentric contraction condition.

## Materials and Methods

### Subjects

Twelve healthy young men (mean ± standard deviation, 24.2 ± 3.2 years; height, 1.73 ± 0.05 m; body mass, 68.1 ± 11.0 kg) voluntarily participated in the present study. The purpose and risks of this study were explained to each volunteer, and written informed consent was obtained. The Ethics Committee on Human Research of Ritsumeikan University approved this study (IRB-2013-14).

### General experimental setup

**Fig 1:**
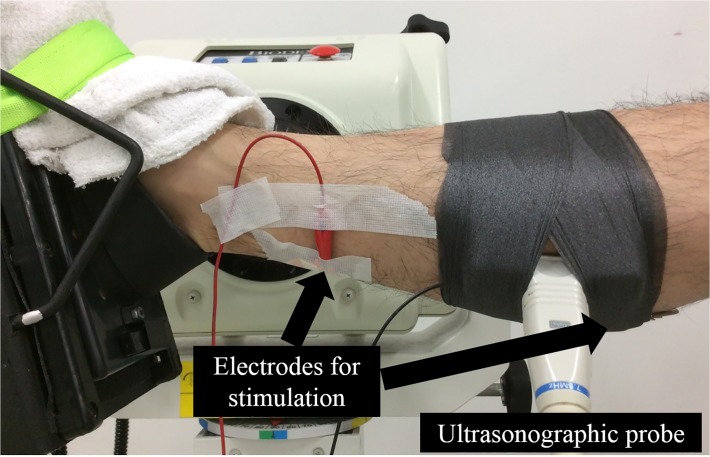
Experimental setup.

In this study, plantarflexion (PF) with a dynamometer (Biodex; SAKAImed, Tokyo, Japan) was adopted as the tested motion, with knee and hip joints flexed at 0° and 80°, respectively (Fig. 1), because similar movement (i.e., ankle hopping with knee joint extended) was adopted in previous studies. [4, 9] The following three conditions were tested: (1) concentric contraction without preliminary contraction (CON), (2) concentric contraction following preliminary eccentric contraction (ECC), and (3) concentric contraction following preliminary isometric contraction (ISO). Velocity of eccentric contraction was set at 60°/s, while that of concentric contraction was set at 90°/s. All muscle contractions were evoked by electrical stimulation (SEN-3401; Nihon Kohden, Tokyo, Japan). Ultrasonographic measurement (SSD-3500; Aloka, Tokyo, Japan) was performed simultaneously during the above three trials to examine whether changes in architectural characteristics (i.e., fascicle length and pennation angle) are related to SSC effect.

### Motion control of the dynamometer and settings of electrical stimulation

**Fig 2:**
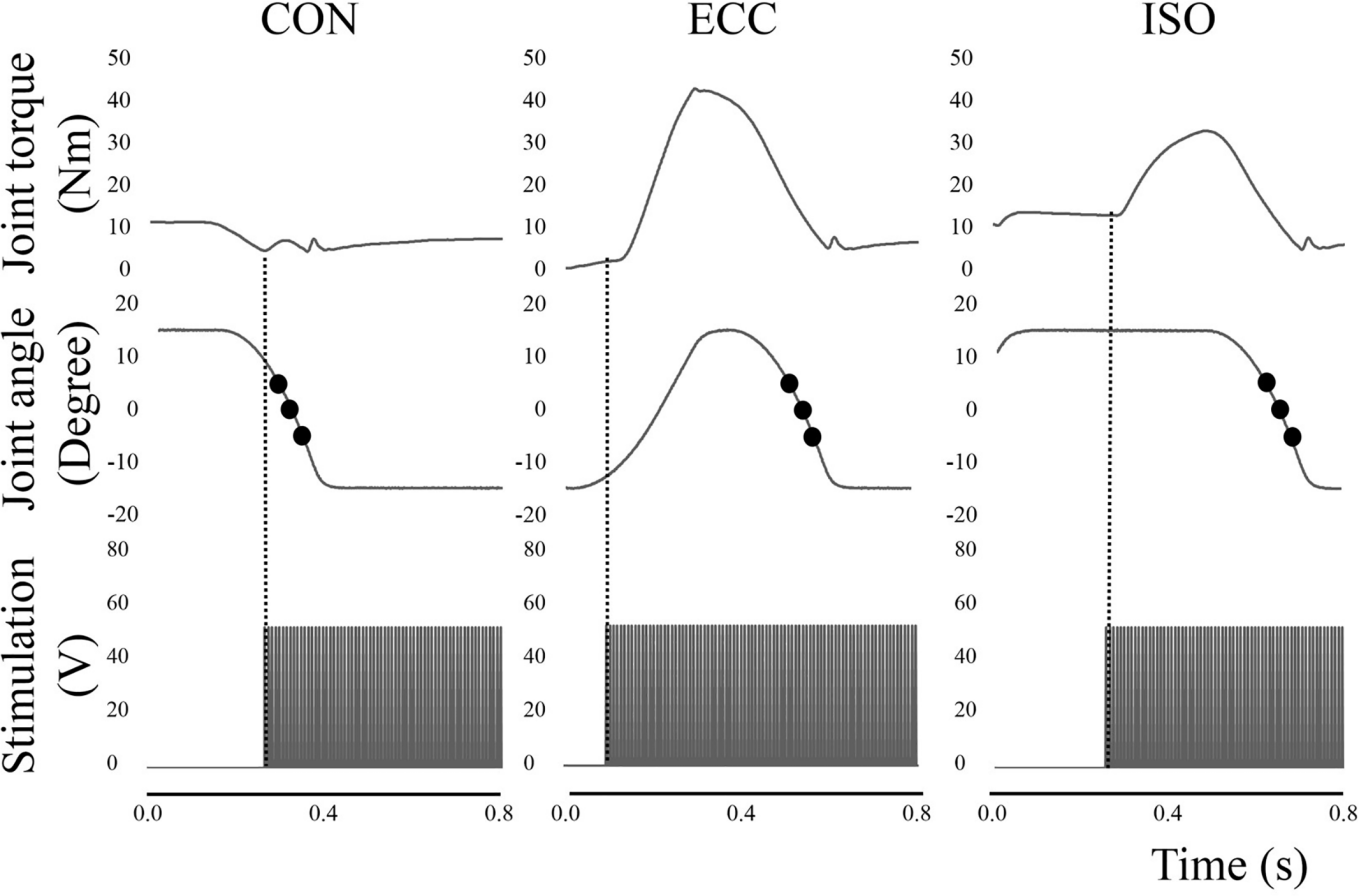
Time course changes in joint torque, joint angle, and timing of electrical stimulation in three contraction conditions. The vertical dotted line shows the onset of electrical stimulation. The filled circles show the timing of joint toque measurement (DF5°, PF0°, and PF5°, respectively). CON; concentric contraction without preliminary contraction, ECC; concentric contraction after preliminary eccentric contraction, ISO; concentric contraction after preliminary isometric contraction, DF; dorsiflexion, PF; plantarflexion.

Typical time course changes of the three types of contraction in this experiment are shown in Fig. 1. Joint motions were controlled by the dynamometer, and range of motion was set from 15° dorsiflexion (DF) to 15° PF. Muscle contractions were evoked by muscle belly stimulation. An anode (4 × 5 cm) was placed on the proximal side of the triceps surae, while a cathode (4 × 5 cm) was placed on the distal side of the soleus. The parameters of electrical stimulation were as follows: pulse frequency, 100 Hz; pulse duration, 0.5 ms; and train duration, 1.5 s. To determine the intensity of electrical stimulation, maximal voluntary isometric contraction in PF was performed with the ankle joint angle at PF0°. The joint torque recorded in this contraction was set as 100% intensity. The intensity of electrical stimulation was adjusted to evoke 25% intensity at the identical joint angle; this electrical stimulation intensity was applied to all contractions; 25% intensity was adopted because some subjects could not tolerate a higher intensity (over 30%). In CON condition, to evoke concentric contraction without preliminary contraction, electrical stimulation was applied at the instance when the ankle joint angle passed DF10° in the shortening phase (i.e., from DF15° to PF15°) (left waveform in Fig. 2). In ECC condition, electrical stimulation was applied at the instance when the ankle joint angle passed PF10° in the lengthening phase (i.e., from PF15° to DF15°) (central waveform in Fig. 2). In ISO condition, different from the other conditions, the position of the dynamometer was kept at DF15°. Timing of electrical stimulation was 0.4 s before the dynamometer moved from DF15° to PF15° (right waveform in Fig. 2). These muscle contractions were conducted in a random order, with a rest interval of at least 1 minute between contractions. The sequence of joint torques recorded at the instance of DF5°, PF0°, and PF5° were used in the following analyses. Joint torque and joint angle were recorded with a sampling frequency of 4,000 Hz (Power lab 16/30; ADInstruments, Bella Vista, Australia).

### Ultrasonographic measurement

**Fig 3:**
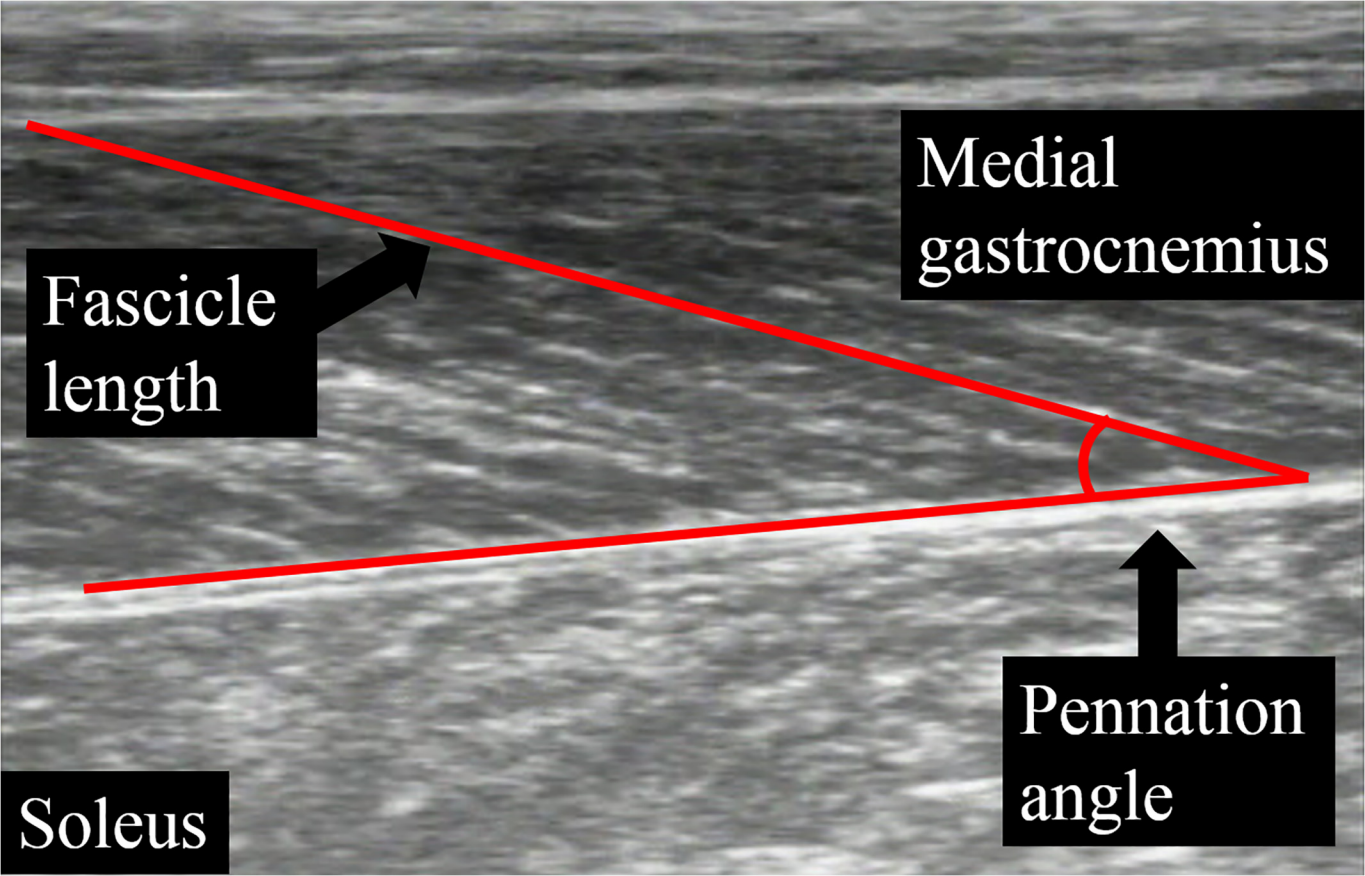
Analyses of fascicle length and pennation angle.

Ultrasonography with a linear array probe (7.5 MHz, Field of view: 6 cm × 6 cm, UST-5710; Aloka, Tokyo, Japan) was used to obtain images of the muscle belly of the medial gastrocnemius. The ultrasonographic probe was fixed onto the skin by using underwrap and surgical tape. Fascicle length and pennation angle were obtained at DF5°, PF0°, and PF5°, which corresponded to the joint torque measurements. Fascicle length was defined as the distance between the intersection composed of the superficial aponeurosis and fascicle and the intersection composed of the deep aponeurosis and fascicle, while pennation angle was defined as the angle composed of the fascicle and deep aponeurosis (Fig. 3). Sampling frequency of ultrasonography was set at 30 Hz. Synchronization of joint torque and joint angle was done by inserting a pulse into the ultrasonographic recoding machine. Acquired images were analyzed by Image J 1.47v software (National Institutes of Health, Bethesda, MD, US).

### Analyses and measurements

Joint torques recorded at DF5°, PF0°, and PF5° in ECC and ISO conditions were expressed as relative values with respect to those in CON. This value was defined as the magnitude of SSC effect. In this study, increase in joint torque in the ISO condition was also called SSC effect, though there was no elongation phase in the ISO condition. In this study, all trials were conducted twice, and mean values were adopted for the following analyses to reduce the influence of random error. Coefficient of variation of joint torque, fascicle length, and pennation angle obtained at PF0° in CON condition were 2.3%, 1.1%, and 1.6%, respectively, while the intraclass correlation of those were 0.995, 0.993, and 0.989, respectively.

### Statistics

Two-way analysis of variance (ANOVA) with repeated measures was adopted to examine the interaction (condition × joint angle) and main effect (condition and joint angle) of joint torque, SSC effect, pennation angle, and fascicle length. If the interaction was significant, one-way ANOVA with repeated measures followed by subsequent post-hoc testing (Bonferroni’s correction) was conducted. Effect size for ANOVA was calculated as the partial *η* ^2^. Statistical analyses were performed using SPSS version 20 software (IBM, Tokyo, Japan), with the level of statistical significance set at *P* < 0.05

## Results

**Fig 4:**
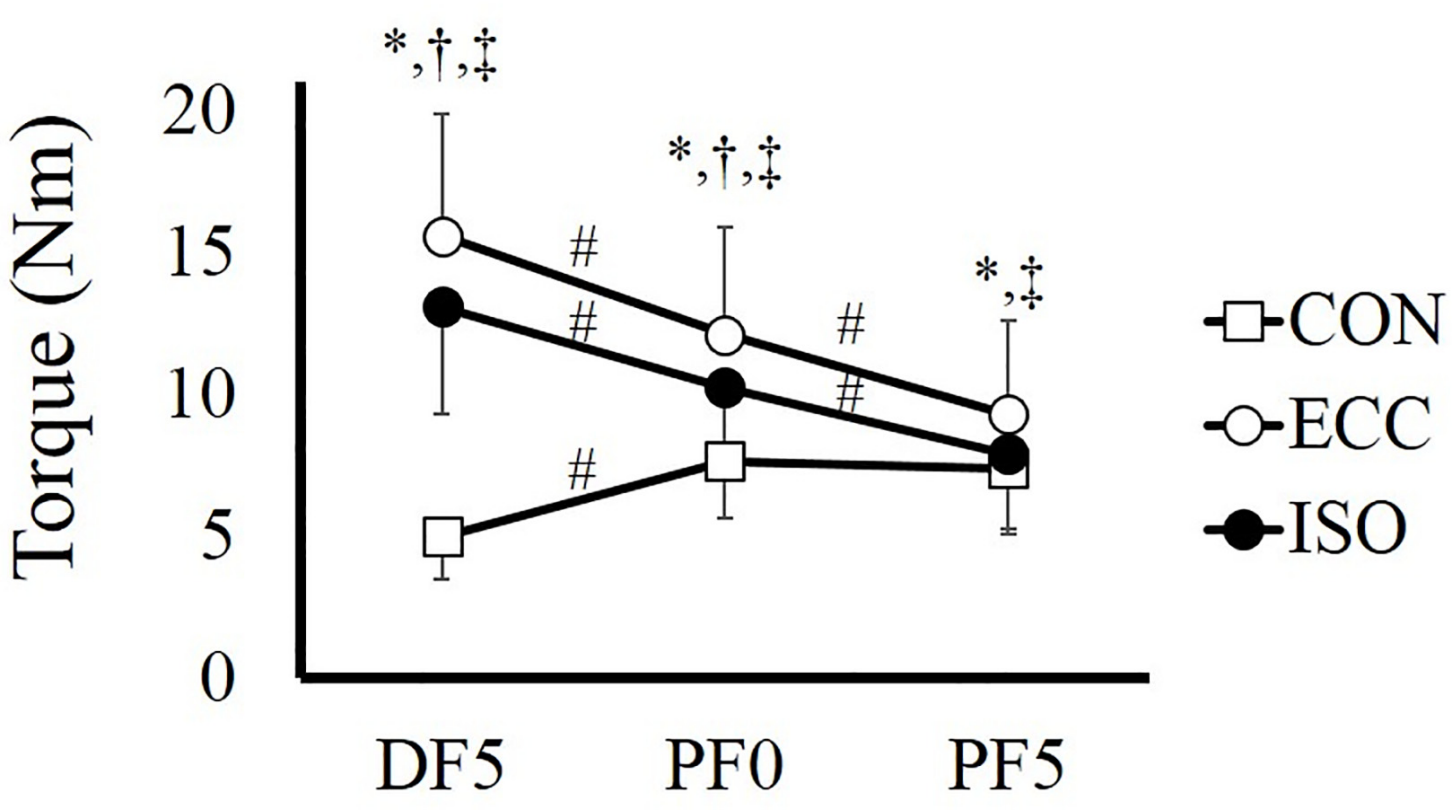
Joint torque recorded at DF5°, PF0°, and PF5° in CON, ECC, and ISO conditions. # indicates a significant difference among joint angles in each contraction condition (*P* < 0.05). * indicates a significant difference between CON and ECC at each joint angle (*P* < 0.05). † indicates a significant difference between CON and ISO at each joint angle (*P* < 0.05). ‡ indicates a significant difference between ECC and ISO at each joint angle (*P* < 0.05). CON; concentric contraction without preliminary contraction, ECC; concentric contraction after preliminary eccentric contraction, ISO; concentric contraction after preliminary isometric contraction, DF; dorsiflexion, PF; plantarflexion.

For joint torque, two-way ANOVA with repeated measures revealed a significant interaction (*F* = 124.988, partial *η* ^2^ = 0.919, *P* < 0.001). Subsequent analyses showed that force was significantly larger in the order corresponding to ECC, ISO, and CON conditions at DF5° (*P* < 0.001–0.003) and PF0° (*P* < 0.001–0.009), whereas force was not different between ISO and CON conditions at PF5° (*P* = 0.751). In addition, force decreased significantly in the order corresponding to DF5°, PF0°, and PF5° in ECC (*P* < 0.001) and ISO (*P* < 0.001) conditions. However, in CON condition, force was higher at PF0° than at DF5° (*P <* 0.001), and was not different between PF0° and PF5° (*P* > 0.999) (Fig. 4).

**Fig 5:**
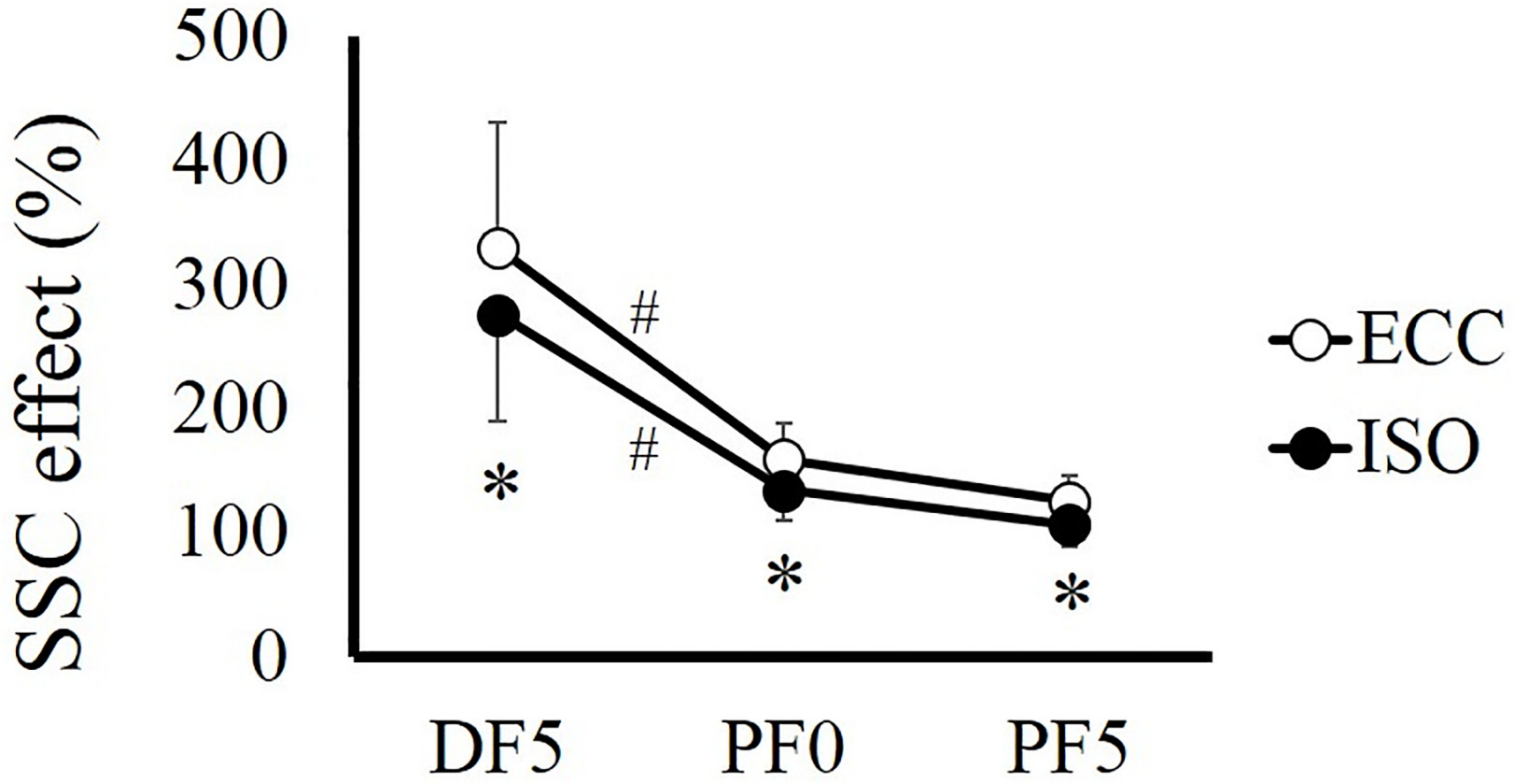
SSC effect calculated at DF5°, PF0°, and PF5° in ECC and ISO conditions. # indicates a significant difference among joint angles in each contraction condition (*P* < 0.05). * indicates a significant difference between ECC and ISO at each joint angle (*P* < 0.05). CON; concentric contraction without preliminary contraction, ECC; concentric contraction after preliminary eccentric contraction, ISO; concentric contraction after preliminary isometric contraction, DF; dorsiflexion, PF; plantarflexion.

For SSC effect, a significant interaction was found (*F* = 3569.482, partial *η* ^2^ = 0.689, *P* < 0.001). Subsequent analyses showed that SSC effect was significantly larger in ECC condition than in ISO condition at all joint angles (*P* < 0.001). In addition, SSC effect was significantly larger at DF5° than at the other two joint angles in both conditions (*P* < 0.001), whereas no significant difference was found between PF0° and PF5° in ECC (*P* = 0.328) and ISO (*P* = 0.377) conditions (Fig. 5).

**Fig 6:**
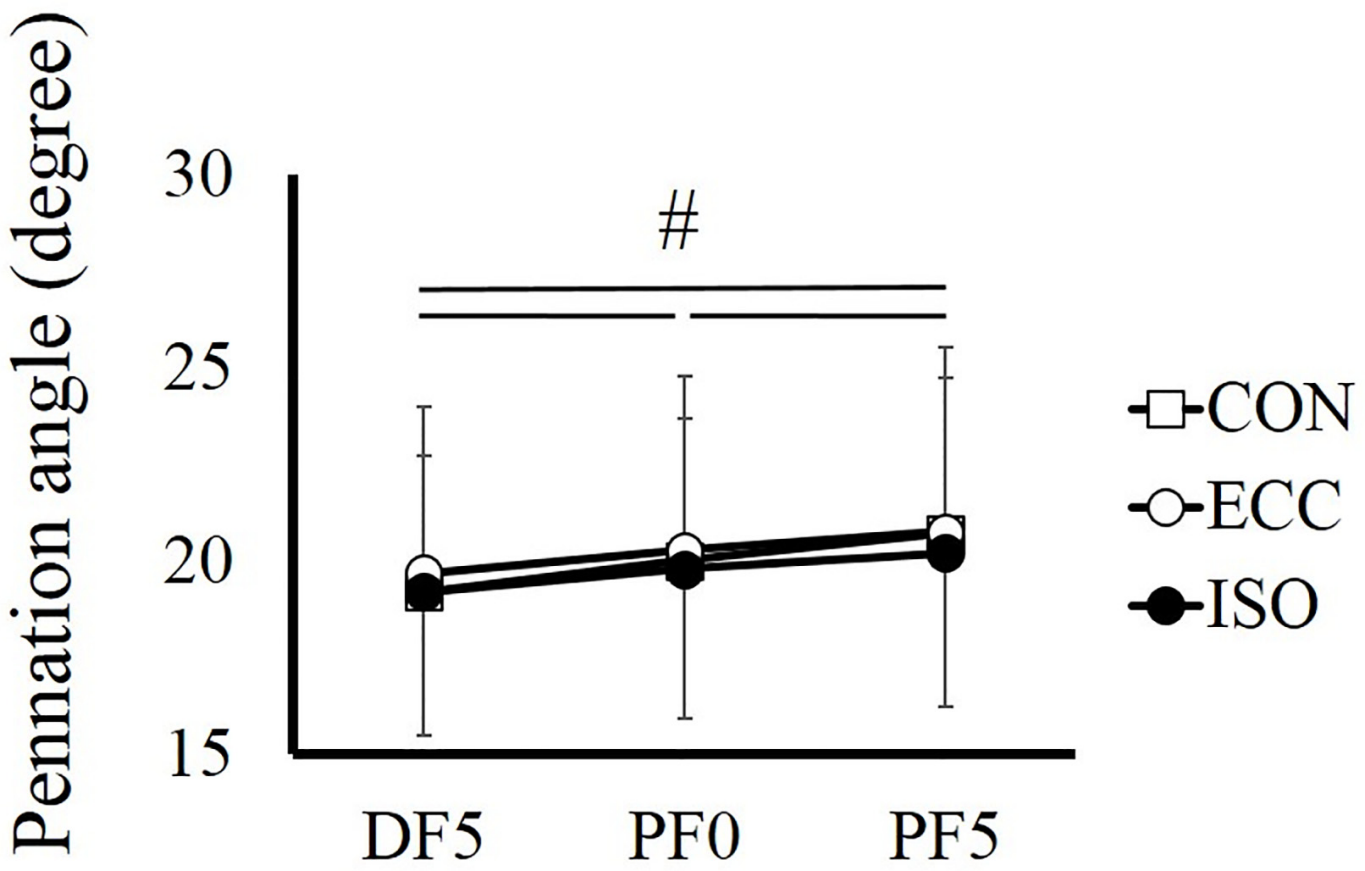
Pennation angle recorded at DF5°, PF0°, and PF5° in CON, ECC, and ISO conditions. # indicates a significant difference among joint angles (*P* < 0.05). CON; concentric contraction without preliminary contraction, ECC; concentric contraction after preliminary eccentric contraction, ISO; concentric contraction after preliminary isometric contraction, DF; dorsiflexion, PF; plantarflexion.

For pennation angle, no significant interaction was found (*F* = 1.664, partial *η* ^2^ = 0.131, *P* = 0.176), and a main effect was found only in joint angle (*F* = 40.325, partial *η* ^2^ = 0.786, *P* < 0.001). Pennation angle increased significantly as the ankle joint plantarflexed (*P* < 0.001) (Fig. 6).

**Fig 7:**
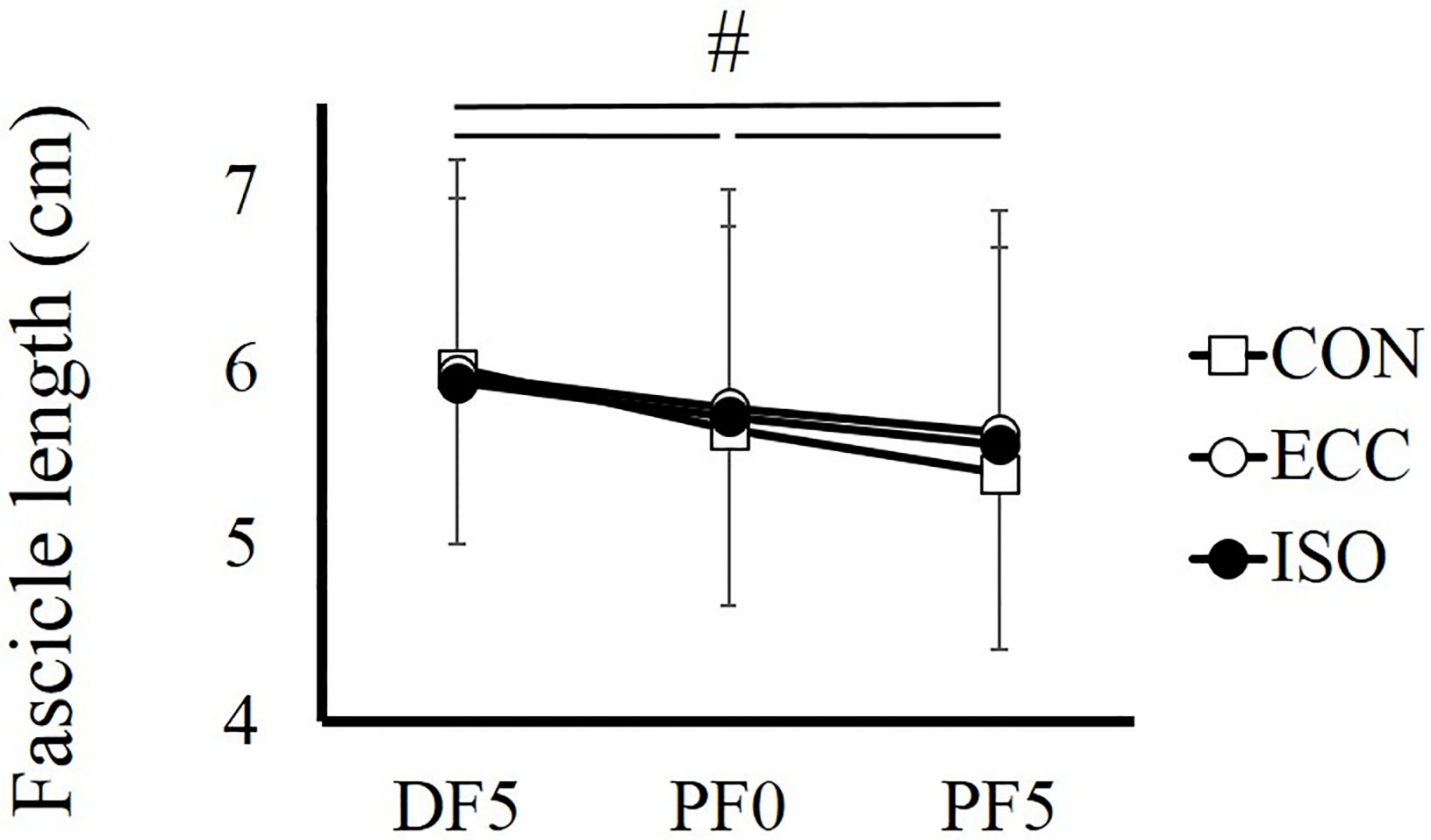
Fascicle length recorded at DF5°, PF0°, and PF5° in CON, ECC, and ISO conditions. # indicates a significant difference among joint angles (*P* < 0.05). CON; concentric contraction without preliminary contraction, ECC; concentric contraction after preliminary eccentric contraction, ISO; concentric contraction after preliminary isometric contraction, DF; dorsiflexion, PF; plantarflexion.

For fascicle length, a significant interaction was found (*F* = 7.442, partial *η* ^2^ = 0.404, *P* = 0.001). Additional analyses showed that fascicle length decreased significantly as the ankle joint plantarflexed (*P* < 0.001). On the other hand, there was no significant difference among conditions (*P* > 0.213–0.999) (Fig. 7).

## Discussion

In this study, we examined in detail the factors that contributed to the SSC effect in vivo by isolating the influence of preactivation by adopting preliminary isometric contraction in addition to preliminary eccentric contraction. As a result, although there was no active lengthening phase in ISO condition (i.e., there was no tendon elongation or residual force enhancement), a remarkable SSC effect was confirmed, especially in the fast half of motion. Thus, preactivation is strongly related to the SSC effect. The result that the SSC effect was larger in the ECC condition than in the ISO condition indicates that tendon elongation and/or residual force enhancement contributes to SSC effect. However, the extent of contribution of these factors would be small compared with that of the contribution of preactivation because the extent of difference in SSC effect was smaller (329% in ECC, 276% in ISO, at DF5°) than the contribution of SSC effect confirmed in the ISO condition (276% in DF5°). These data indicate that the SSC effect of 176% was caused by preactivation and that SSC effect of 53% was caused by tendon elongation and/or residual force enhancement in the current study.

We confirmed that preactivation has a substantial influence on SSC effect by adopting a condition that included preactivation but did not include stretch reflex, tendon elongation, or residual force enhancement. The results were in line with those of previous studies [17, 20, 21]. The reason why the contribution of preactivation is large can be explained by the joint torque development phase in concentric contraction without preliminary contraction. The human medial gastrocnemius, adopted in the current study, operates in the ascending limb according to the force-length relationship [22, 23]. Thus, as the ankle joint angle flexes, muscle force should decrease according to the force-length relationship [24]. Indeed, joint torque decreased as the ankle joint angle flexed in ISO and ECC conditions. However, joint torque increased as the ankle joint angle flexed in CON condition, even in the ascending limb. This phenomenon indicates that activation level in this phase was still submaximal. Because it takes several times to develop muscle force from 0% to 100% [25, 26], activation level is submaximal in the first phase of muscle contraction. Thus, if this delay can be avoided by preactivation, joint torque attained in concentric contraction is increased, especially in the first phase of contraction. Indeed, the current study confirmed that the extent of SSC effect was much larger in the first phase of concentric contraction (i.e., DF5°) in both conditions. Taken together, preactivation has a critical influence on SSC effect.

We confirmed that SSC effect was larger in ECC condition than in ISO condition at all joint angles. Thus, active lengthening–induced force potentiation (i.e., tendon elongation and/or residual force enhancement) also contributed to SSC effect. Tendon elongation affects changes in muscle length [9, 27], which in turn, modulates the force-generating capability of muscle. For example, if the tendon elongates during the eccentric phase and shortens during the subsequent concentric phase, instead of the muscle, the muscle can operate almost isometrically, which enhances the force-generating capability of the muscle due to the force-velocity relationship [28]. However, a recent study reported that the extent of tendon elongation was small [29]. Thus, it may be difficult to stretch the tendon substantially, instead of the muscle, to prevent muscle lengthening and shortening. Indeed, in the current study, although joint torque was clearly larger in ECC condition than in ISO condition during the preliminary contraction phase, Achilles tendon length during the concentric contraction phase was similar between ISO and ECC conditions (judging from the results that changes in fascicle length (muscle length) and joint angle (muscle-tendon complex length) were similar between both conditions). These results indicate that the tendon did not elongate as a function of muscle force, at least in the current experimental condition. Taken together, the extent of tendon elongation would be small or negligible in this experiment, suggesting that the contribution of tendon elongation to SSC effect would be small.

Considering the aforementioned relationship between tendon elongation and SSC effect, the other factor, residual force enhancement, would be a more reasonable explanation for the different SSC effect between ECC and ISO conditions. Several studies have examined the influence of residual force enhancement on SSC effect and reported consistent results with the current study that residual force enhancement does contribute to SSC effect [1, 12, 14]. Although the mechanism of residual force enhancement has been debated [30]—that is, whether it is caused by Ca^2+^-induced changes in titin filament stiffness [31, 32] and/or sarcomere non-uniformity [33, 34]—this phenomenon is well known and frequently confirmed in isolated whole muscle [35], isolated single-muscle fiber [36], myofibril [37], and human whole muscle [38]. Thus, residual force enhancement can be explained by active lengthening–induced force potentiation (i.e., SSC effect) in this study. Regrettably, it is difficult to further clarify the precise relationship between SSC effect and residual force enhancement from the current data. One way to examine the aforementioned relationship is to change muscle length during SSC, since residual force enhancement is related to sarcomere length and magnitude of elongation [13, 36].

Ultrasonographic measurement revealed that architectural characteristics (i.e., pennation angle and fascicle length) were similar between ISO and ECC conditions. These results indicate that active lengthening–induced force potentiation is not due to the morphologic properties of muscle. We have to point out that sampling frequency of ultrasonographic images in the current study (i.e., 30 Hz) was relatively low, which may have led to measurement error of fascicle length and/or pennation angle. However, the influence of the error caused by low-sampling frequency may have been small because the repeatability of fascicle length and pennation angle measurements between two of the same trials was very high (coefficient of variation of fascicle length and pennation angle obtained at PF0° in CON condition were 1.1% and 1.6%, respectively, while intraclass correlation of those were 0.993 and 0.989, respectively).

In this study, the influence of stretch reflex, which is considered one of the factors of the SSC effect [5, 6], was ruled out by using electrical stimulation instead of voluntary activation. Thus, we could not confirm whether stretch reflex contributes to SSC effect in this study. Previous studies examining the influence of stretch reflex on SSC effect reported that increased electromyographic activity was not observed after eccentric contraction [17]. These results would be caused by the saturation effect of stretch reflex. The influence of the stretch reflex decreases as contraction intensity increases [39]. Thus, stretch reflex would not contribute to SSC effect, especially when contraction intensity is high, such as in vertical jump with maximal effort.

In summary, we examined in detail the factors contributing to SSC effect in vivo. As a result, preactivation had a remarkable influence on SSC effect, which was confirmed by the magnitude of SSC effect in the condition without active lengthening. In addition, active lengthening–induced force potentiation (i.e., tendon elongation and/or residual force enhancement) also contributed to SSC effect. This was confirmed by a comparison of the SSC effect between conditions with active lengthening (ECC) and those without active lengthening (ISO). Active lengthening–induced force potentiation may be attributable to residual force enhancement because the magnitude of tendon elongation would be small even in the condition with active lengthening contraction.
